# Mechanical and Durability Properties of Cementless Concretes Made Using Three Types of CaO-Activated GGBFS Binders

**DOI:** 10.3390/ma15010271

**Published:** 2021-12-30

**Authors:** Woo Sung Yum, Juan Yu, Dongho Jeon, Haemin Song, Sungwon Sim, Do Hoon Kim, Jae Eun Oh

**Affiliations:** School of Urban and Environmental Engineering, Ulsan National Institute of Science and Technology (UNIST), UNIST-gil 50, Ulju-gun, Ulsan 44919, Korea; reikoku@nate.com (W.S.Y.); cp9eins@unist.ac.kr (J.Y.); dhjeon0707@gmail.com (D.J.); haemin93@unist.ac.kr (H.S.); conor123@naver.com (S.S.); elvis03@unist.ac.kr (D.H.K.)

**Keywords:** CaO-activation, auxiliary activator, GGBFS, chemical resistance, freezing, thawing

## Abstract

This study examined the mechanical and durability properties of CaO-activated ground-granulated blast-furnace slag (GGBFS) concretes made with three different additives (CaCl_2_, Ca(HCOO)_2_, and Ca(NO_3_)_2_) and compared their properties to the concrete made with 100% Ordinary Portland Cement (OPC). All concrete mixtures satisfied targeted air content and slump ranges but exhibited significantly different mechanical and durability properties. The CaO-activated GGBFS concretes showed different strength levels, depending on the type of additive. The added CaCl_2_ was the most effective, but Ca(NO_3_)_2_ was the least effective at increasing mechanical strength in the CaO-activated GGBFS system. The OPC concrete showed the most excellent freezing–thawing resistance in the durability test, but only the CaO-activated GGBFS concrete with CaCl_2_ exhibited relatively similar resistance. In addition, the chemical resistance was significantly dependent on the type of acid solution and the type of binder. The OPC concrete had the best resistance in the HCl solution, while all CaO-activated GGBFS concretes had relatively low resistances. However, in the H_2_SO_4_ solution, all CaO-activated GGBFS concretes had better resistance than the OPC concrete. All concrete with sulfate ions had ettringite before immersion. However, when they were immersed in HCl solution, ettringite tended to decrease, and gypsum was generated. Meanwhile, the CaO-activated GGBFS concrete with CaCl_2_ did not change the type of reaction product, possibly due to the absence of ettringite and Ca(OH)_2_. When immersed in an H_2_SO_4_ solution, ettringite decreased, and gypsum increased in all concrete. In addition, the CaO-activated concrete with CaCl_2_ had a considerable amount of gypsum; it seemed that the dissolved C-S-H and calcite, due to the low pH, likely produced Ca^2+^ ions, and gypsum formed from the reaction between Ca^2+^ and H_2_SO_4_.

## 1. Introduction

With global warming, abnormal climates are occurring worldwide, causing many problems [1,2]. The international community agreed to reduce greenhouse gas emissions through the Paris Climate Agreement, to prevent accelerating global warming.

There is increasing demand for materials to replace Portland cement [3,4] because the OPC industry accounts for 8 to 10% of worldwide CO_2_ emissions, accelerating global warming [5]. Therefore, many studies have been conducted on developing alternative binders with a low carbon footprint, using various industrial by-products, such as fly ash or ground-granulated blast-furnace slag (GGBFS) to replace OPC [6,7,8]. Fly ash-based geopolymers using fly ash as the main material use alkaline activators, such as sodium hydroxide (NaOH), potassium hydroxide (KOH), or sodium silicates (e.g., water glass). Palomo et al. [9] reported that geopolymer activated with NaOH 8–12 M cured at 85 °C showed a compressive strength of about 40 MPa, and it reached nearly 90 MPa when water glass was added to the binder. Sun et al. [10] explored the effect of three different aggregate sizes on the strength of geopolymers. M.A.M Ariffin et al. [11] showed that geopolymer concrete exhibited better sulfuric acid resistance than OPC concrete. Although geopolymer has high compressive strength and durability, it has the disadvantage that its performance changes depending on the type of activator and curing temperature [12]. Furthermore, U Yurt et al. [13] showed the performance of alkali-activated GGBFS composite varying with the different alkali activators and activation temperature. In addition, many studies [14,15] reported that alkaline activated GGBFS binders have high compressive strength and chemical resistance. However, similar to geopolymers, GGBFS-based cementless binders have drawbacks in the following usages [16]: (1) high material costs, (2) user safety, and (3) difficulty in producing a one-part binder. Despite these problems, alkali-activated binders produce comparable mechanical properties compared to the OPC binder [17,18]; thus, many studies have been conducted at the concrete level to validate the mechanical and durability properties [19,20].

On the other hand, the CaO-activated binder system [21,22] relatively overcame the disadvantages mentioned above of alkali-activated binders. Kim et al. [23] studied CaO-activated GGBFS binder, and Yum et al. [21,24,25] enhanced mechanical properties of CaO-activated GGBFS binders by adding different types of additional activators. In addition, there are studies to develop cementless binders by activating fly ash using CaO and other chemical additives [22,26]. Although the CaO-activated binders can potentially apply to construction fields, no study has been conducted at the concrete level on mechanical properties and durability. 

Therefore, this study compared the mechanical and durability properties of various CaO-activated GGBFS concretes using additives, CaCl_2_, Ca(HCOO)_2_, and Ca(NO_3_)_2_. Mechanical properties, such as compressive strength, tensile splitting strength, and elastic modulus were measured. In addition, the durability properties were evaluated by the freezing and thawing cycle test and the chemical resistance test using hydrochloric acid (HCl) and sulfuric acid (H_2_SO_4_). Changes in hydration products were examined through powder X-ray diffraction (XRD) measurement and thermogravimetry analysis (TGA).

## 2. Materials and Methods

Commercial OPC (type I, 42.5N) and GGBFS were used in this study. In addition, all used chemicals were analytical grade. CaO (Daejung, Korea) was the main activator, and CaCl_2_ (Daejung, Korea), Ca(HCOO)_2_ (Aladdin, China), Ca(NO_3_)_2_ (Sigma Aldrich, Burlington, MA, USA), and CaSO_4_ (Daejung, Korea) were additives. The maximum size of coarse aggregate was 25 mm. Fine aggregate were classified as aggregates with a size less than 5 mm. All aggregates were used under saturated surface dry (SSD) conditions. In addition, a naphthalene-based commercial plasticizer was used in concrete mixtures.

XRD, XRF, and particle size distribution analysis were performed to identify the characteristics of the raw materials (i.e., OPC and GGBFS). XRD patterns were recorded by means of a D/Max2500 V diffractometer (Rigaku, Japan) with an incident beam of Cu-kα radiation (λ = 1.5418 nm) in a 2θ scanning range from 5° to 60°; X-ray fluorescence was conducted with S8-Tiger spectrometer (Bruker, Billerica, MA, USA). 

**Figure 1:**
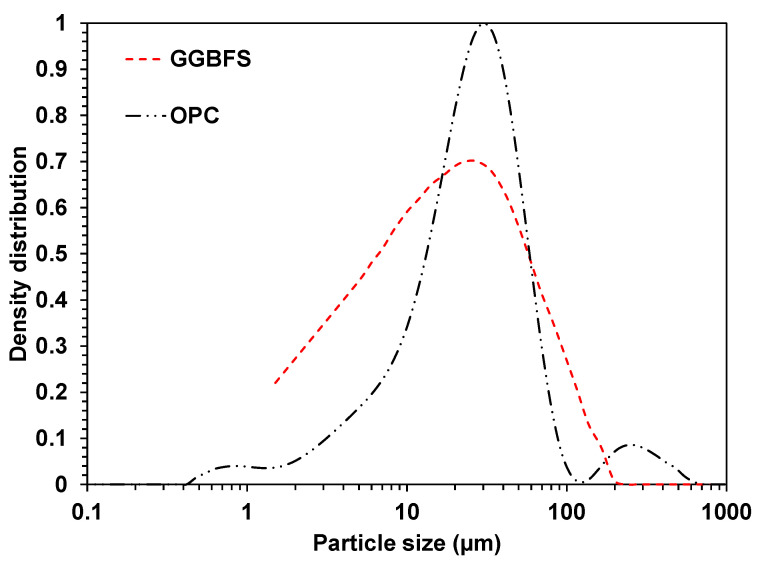
Particle size distributions of OPC and GGBFS.

**Figure 2:**
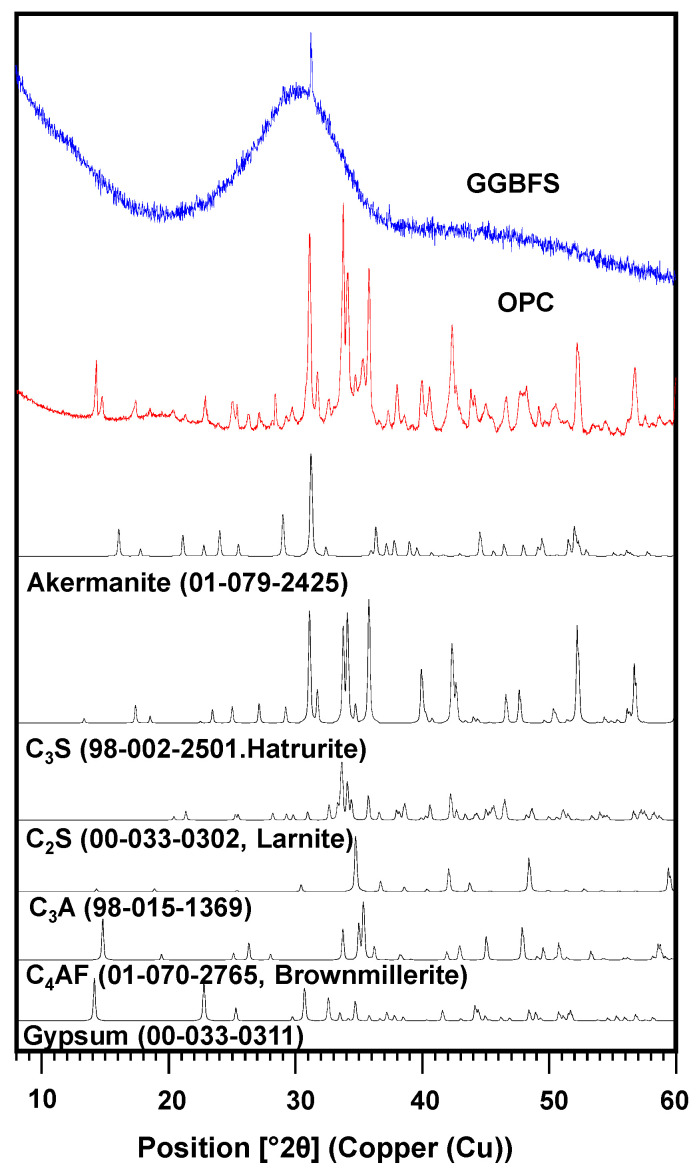
XRD patterns of OPC and GGBFS with identified phases.

Particle size distribution results (Figure 1) show that the average particle sizes of OPC and GGBFS were 25 and 30 µm, respectively. Table 1 exhibits the XRF results of OPC and GGBFS, and they mainly consisted of CaO and SiO_2_. In Figure 2, the XRD results show that akermanite [Ca_2_Mg(Si_2_O_7_)] was identified in GGBFS, and GGBFS primarily consisted of an amorphous phase. On the other hand, dicalcium silicate (C_2_S), tricalcium silicate (C_3_S), tetracalcium aluminoferrite (C_4_AF), tricalcium aluminate (C_3_A), and gypsum (CaSO_4_) were identified in OPC [27]. 

The material proportions of binders used to produce concrete samples are shown in Table 2. The P-binder was 100% OPC. The other binders (C-, F-, and N-binders) were CaO-activated GGBFS binders, which used CaO as a primary activator for activating GGBFS while included different additives. The C-binder, F-binder, and N-binder used CaCl_2_, Ca(HCOO)_2_, and Ca(NO_3_)_2_, respectively, as additives, and their material proportions were determined via previous studies [21,24,25]. Note that F-binder and N-binder additionally used five weight percentages of CaSO_4_ to enhance the early compressive strength of the binders.

The mixture proportions of concrete are given in Table 3, and the samples were labeled similarly after the used binders. According to the Korea Concrete Specification [28], concrete mixture proportions were designed, and the targeted compressive strength was above 45 MPa. Targeted slump and air content ranges were 5–15 cm and 3–6%, respectively. 

For concrete production, all mixing procedures followed the Korea Standard (KS) F 2403 [29]. The fresh concrete samples were cast in cylindrical molds (φ 10 cm × 20 cm^3^) to test determine compressive strength, split strength, elastic modulus, and chemical resistance, and samples in prism molds (10 × 10 × 40 cm^3^) for freezing and thawing cycle tests. The fresh concrete samples were compacted and cured at 23 °C and 99% relative humidity.

Slump and air content were examined according to the KS F 2402 and KS F 2421 [30,31]. The slump and air content tests were conducted once for each mixture proportion. Triplicate samples were tested for compressive and split strength of each mixture at 3, 7, and 28 days of curing. Chord elastic modulus was measured for the 28-day cured samples, and triplicate specimens were tested for each mixture. The elastic modulus was calculated using the chord modulus using stress-strain curves obtained from three linear variable differential transformers (LVDT) installed in longitudinal directions of concrete [27]. The measured elastic modulus was compared with models proposed by the American Concrete Institute (ACI) 318 and the Euro-International Committee for Concrete—the International Federation for Pre-stressing (CEB-FIP); the CEP-FIP equations are as follows [32,33]:

$$\mathrm{ACI}\;\mathrm{Model}:\;E_{c}=W_{c}{}^{1.5}\times 0.043\sqrt{f_{28}}$$

$$\mathrm{CEB}-\mathrm{FIP}:\;E_{c}=2.15\times 10^{4}{\left(\frac{f_{28}}{10}\right)}^{1/3}$$

where $E_{c}$ is the elastic modulus (GPa), $f_{28}$ is the 28-day compressive strength (MPa), and $w_{c}$ is density (kg/m^3^).

Freezing and thawing cycle resistance tests were performed using 14-day cured samples, and all experimental procedures followed by KS F 2456 [34]. In addition, three identical samples were used on each measurement date. The initial weight and dynamic modulus of elasticity were recorded for 14 days after curing, and then the samples were tested for freezing and thawing cycles. Each cycle consisted of −20 °C to 4 °C; the time of each cycle was less than 4 h, and the relative dynamic modulus of elasticity and weight change were measured every 30 cycles. Three identical samples were used for each concrete, and the experiments were conducted up to 300 cycles. 

Chemical resistance tests were carried out according to ASTM C 267 [35]. The 28-day cured samples were immersed in a container with 5% HCl and 5% H_2_SO_4_ solutions, and the solutions were replaced once in 4 weeks. The weight changes were measured at 3, 7, 14, 28, 56, and 91 days after immersion to identify the evolution of reaction products. On each weight measurement, three identical specimens were weighed. Analysis of XRD and TGA identified reaction products after 28, 56, and 91 days of immersion. The exposed samples were fractured and finely ground using a mortar and pestle. In addition, XRD and TGA were conducted once for each measurement day and mixture proportion. In addition, SDT Q600 (TA Instruments, New Castle, DE, USA) was used to measure the reaction products of each sample with a heating rate of 30 °C/min from 25 to 1000 °C in a nitrogen atmosphere in an alumina pan.

## 3. Results

### 3.1. Slump and Air Content

**Figure 3:**
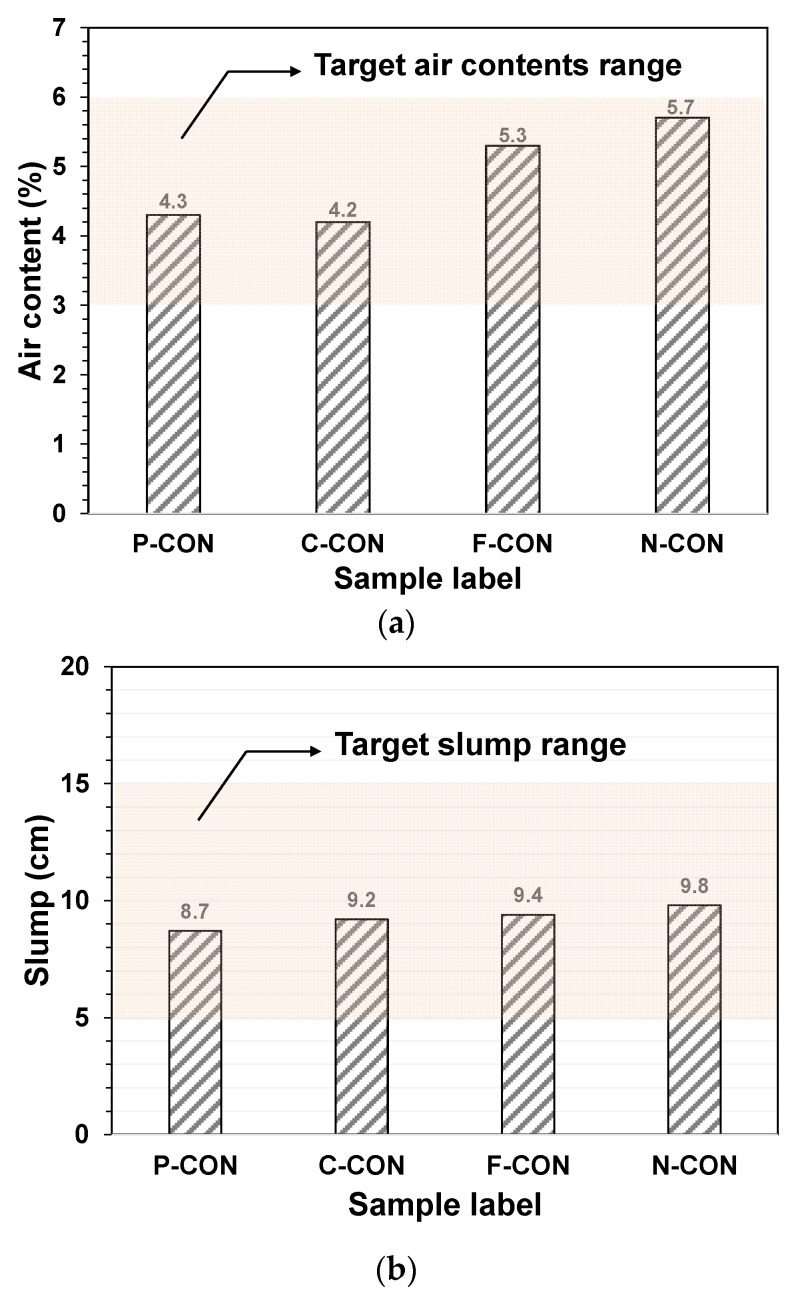
The test results: (**a**) air content and (**b**) slump of fresh concrete samples.

Figure 3 displays the air content and slump measurement test results. The air content and the slump values satisfied the criteria specified in KS F 2421 (air content = 3–6%) and KS F 2402 (slump = 5–15 cm) [30,31], respectively.

### 3.2. Compressive and Split Strength

**Figure 4:**
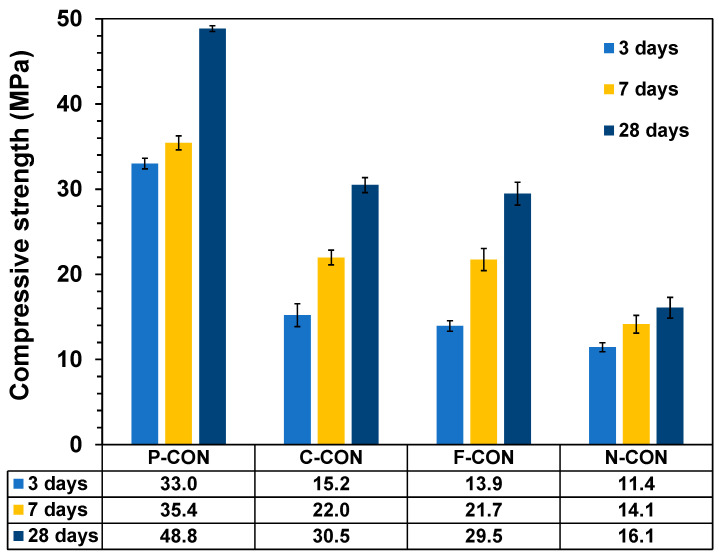
Compressive strengths of hardened concrete samples.

Compressive strengths of hardened concretes are shown in Figure 4, and the greatest compressive strength was measured from P-CON at all curing days. In addition, all mixtures showed increased strengths with increasing curing days. All cementless concrete samples exhibited significantly lower compressive strengths than P-CON at three days, mainly due to the lower reaction rate of CaO-activated GGBFS binders than OPC binder [36]. The type of used additives influenced the strength development of CaO-activated GGBFS concretes. CaCl_2_ was the most effective in increasing strength, while Ca(NO_3_)_2_ was the least effective. 

The mixture proportion of concrete was designed following the Concrete Standard Specification in Korea [37], and the designed 28-day compressive strength (f_cr_) was 45 MPa. The compressive strength was 48 MPa for P-CON, but the CaO-activated GGBFS concretes gained only about 10 to 30 MPa, significantly lower compressive strength than f_cr_. 

**Figure 5:**
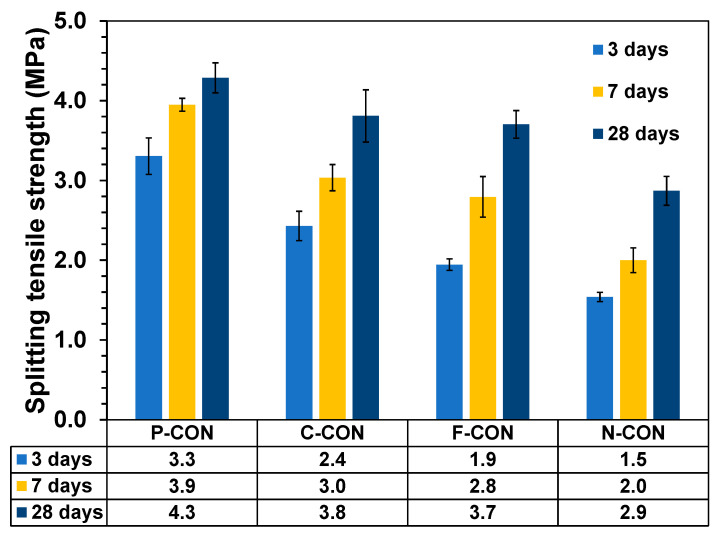
Split strengths of hardened concrete samples.

The split strengths are presented in Figure 5. The tensile strength of OPC concrete was reported as about 10 to 15% of the compressive strength [27]. The concrete specimens made with CaO-activated GGBFS binders having no OPC also exhibited a similar trend in this study.

### 3.3. Chord Elastic Modulus

The measured elastic modulus values of concrete samples are given in Table 4 with the calculated values from ACI 318 and CEB-FIP model codes. It is known that the strength and elastic modulus had a proportional relationship in OPC concrete [27], and the same tendency was observed for all binder types of concrete in this study.

When comparing the experimental values with the calculated values from the ACI and CEB-FIP models, the models showed significantly higher elastic modulus than the measured values. Previous studies similarly reported that the ACI and CEB-FIP models tended to overestimate the elastic modulus of OPC concrete, and it was observed for all types of concretes in this study [38,39,40]. 

### 3.4. Freezing and Thawing Cycle Tests

**Figure 6:**
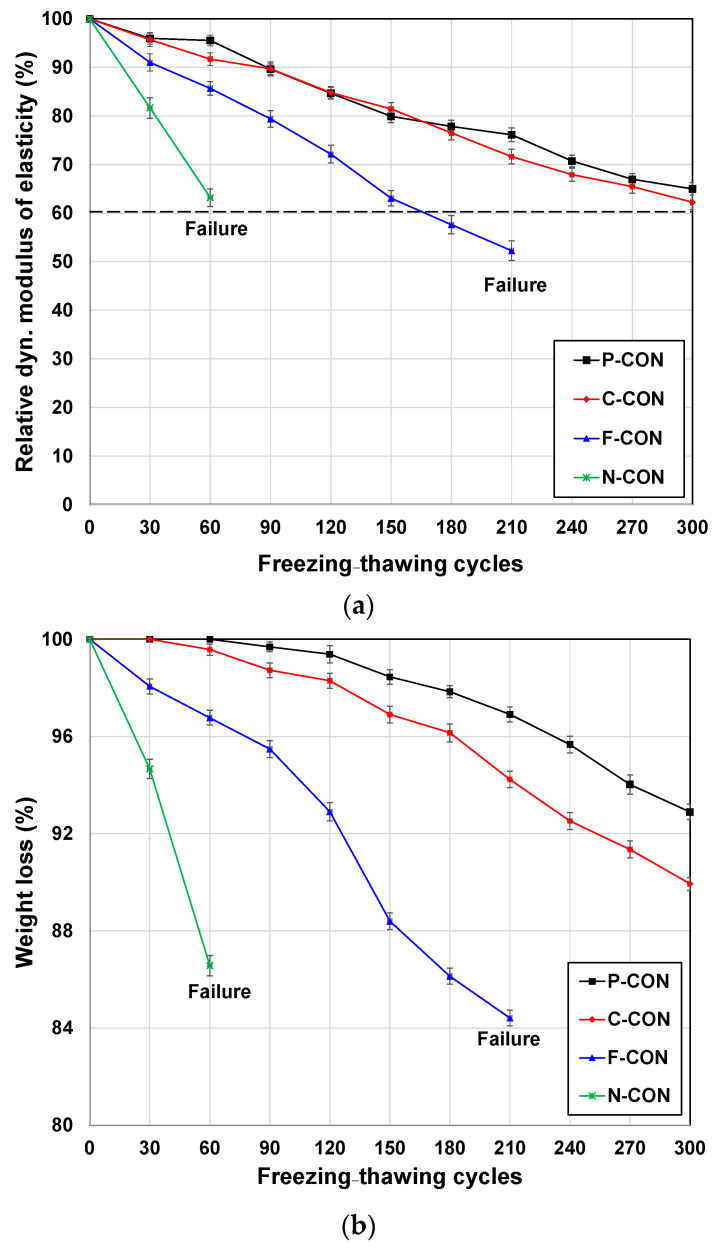
Freezing and thawing test results: (**a**) relative dynamic modulus of elasticity and (**b**) weight loss.

Figure 6 presents the freezing and thawing cycle test results for changing relative dynamic modulus of elasticity and weight loss with increasing cycles. 

The dynamic elastic modulus of P-CON and C-CON decreased down to about 60% up to 300 cycles, while those of F-CON and N-CON were not measured because they were damaged significantly before reaching 300 cycles. Thus, F-CON and N-CON are vulnerable to freezing and thawing cycles.

It is worth noting that although C-CON and F-CON had similar compressive strengths and elastic modulus, they showed significantly different resistances to freezing and thawing cycles. However, a sample with a higher drop of the dynamic modulus tended to show a steeper decrease in the weight. Thus, the strength itself was a less accurate indicator than the dynamic modulus for estimating the ability to resist freezing and thawing cycles in this study.

For OPC concretes, KS F 2456 [34] determines that OPC concrete is susceptible to freezing and thawing cycles when OPC concrete samples are destroyed before 300 cycles or when the dynamic elastic modulus decreases below 60% after 300 cycles. Thus, this study implies that KS F 2456 can also be applied to the CaO-activated GGBFS system. 

### 3.5. Chemical Resistance Tests (5% HCl and 5% H_2_SO_4_)

**Figure 7:**
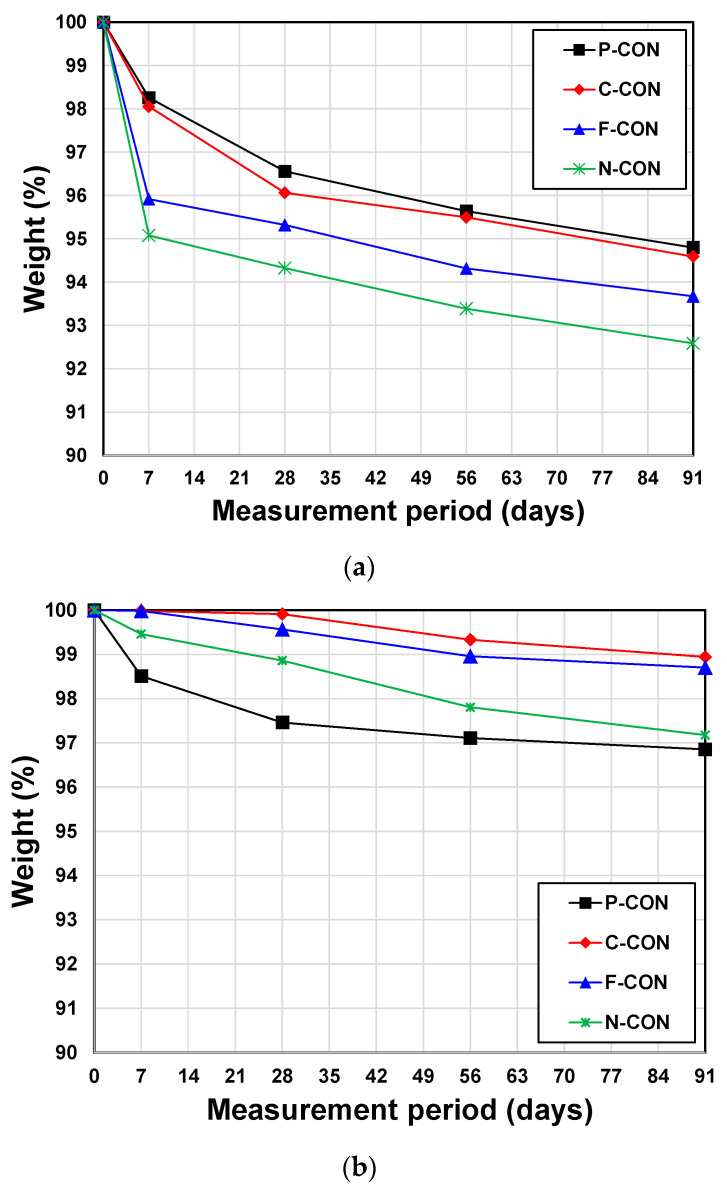
Chemical resistance test results: (**a**) 5% HCl solution and (**b**) 5% H_2_SO_4_ solution.

The results of chemical resistance tests using 5% HCl and 5% H_2_SO_4_ solutions are shown in Figure 7. Significantly different results were obtained, depending on the type of solutions. In 5% HCl aqueous solutions, although weight loss was observed in all immersed samples, P-CON had the most excellent resistance, while N-CON was the most vulnerable. However, the samples immersed in 5% H_2_SO_4_ aqueous solutions yielded different results; the most considerable weight loss occurred in P-CON, followed by N-CON, F-CON, and C-CON, in order.

#### XRD and TG/DTG Analysis in Chemical Resistance Tests

When concrete samples were immersed in acid solutions (i.e., HCl and H_2_SO_4_), the pH of the concrete was lowered, resulting in the change of hydration products. 

**Figure 8:**
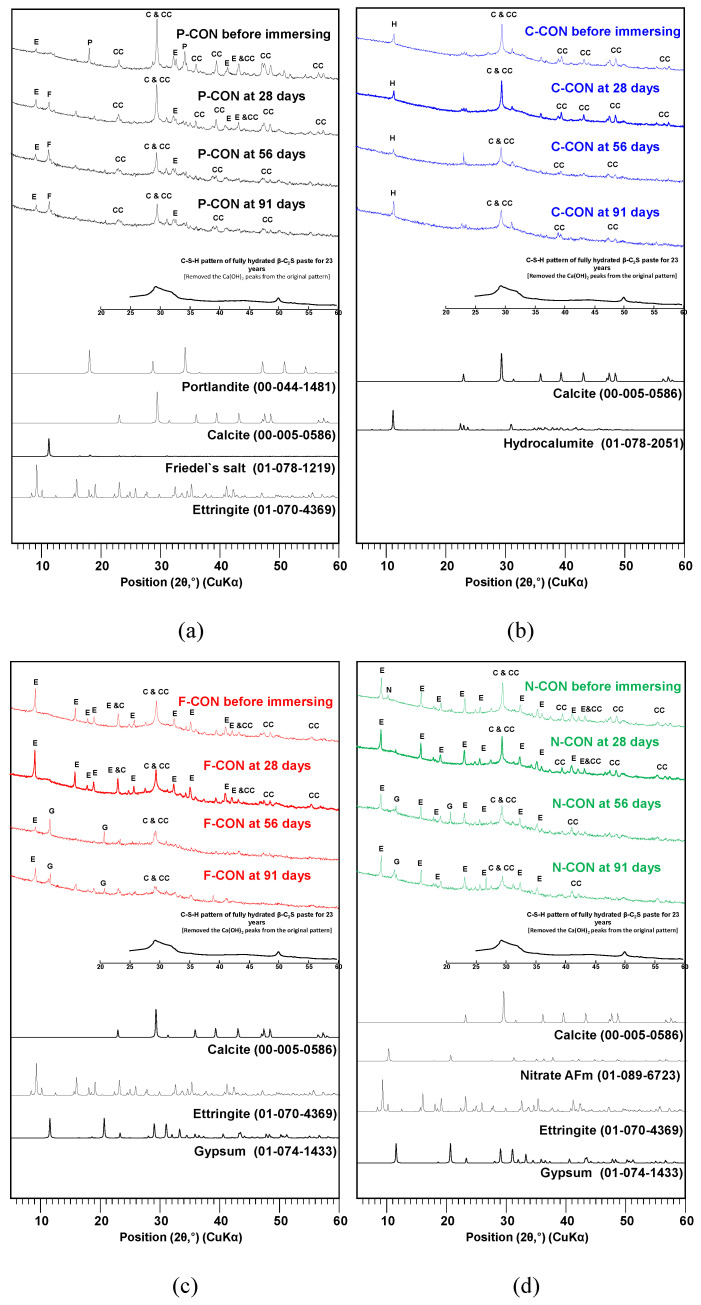
XRD patterns of hardened samples immersed in 5% HCl solution: (**a**) P-CON, (**b**) C-CON, (**c**) F-CON, and (**d**) N-CON. (Note. E: ettringite, C: C-S-H, CC: calcite, G: gypsum, H: hydrocalumite, F: Friedel’s salt, P: portlandite, and N: nitrate AFm, respectively).

**Figure 9:**
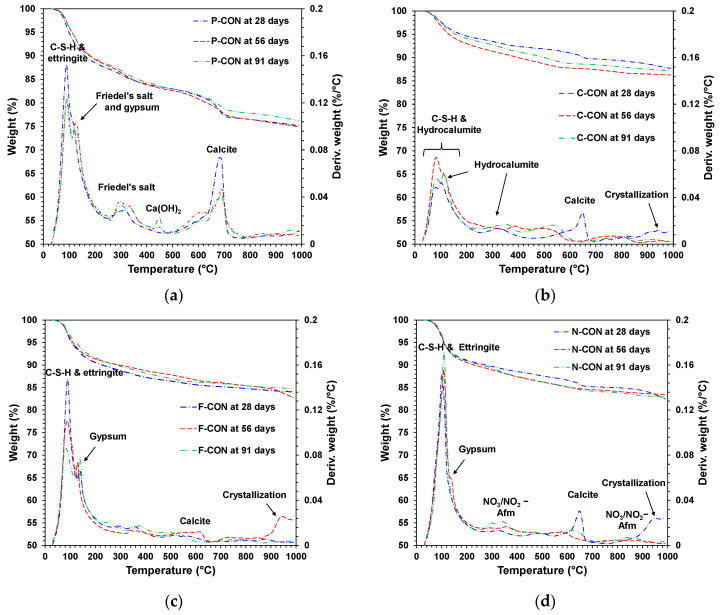
TG/DTG curves of hardened samples immersed in 5% HCl solution: (**a**) P-CON, (**b**) C-CON, (**c**) F-CON, and (**d**) N-CON.

In the HCl solution, the change of the reaction products was investigated using the XRD and TG/DTG analysis, shown in Figure 8 and Figure 9, respectively. After immersion, Ca(OH)_2_ disappeared in the P-CON sample, and Friedel’s salt (Ca_2_Al(OH)_6_Cl(H_2_O)_2_) was found. The HCl solution likely lowered the pH of P-CON, resulting in removing OH^−^ of Ca(OH)_2_ [41,42], and Friedel’s salt was produced by the reaction of Cl^−^ ions from HCl and Ca^2+^ ions from Ca(OH)_2_. In addition, regeneration of Ca(OH)_2_ was observed at the 91-day sample. It seemed that as the chemical resistance test proceeded, Ca(OH)_2_ was regenerated by a complex chemical reaction; more research will likely be needed for the regeneration of Ca(OH)_2_.

The XRD showed that all samples with sulfate ion (SO_4_^2−^) (i.e., P-, F-, and N-CON) had ettringite before immersion. When immersed in HCl solution, the DTG analysis showed that ettringite tended to decrease, and gypsum was generated; F-CON exhibited the most prominent decrease of ettringite, while N-CON displayed the slightest change in ettringite peak. Previous studies [41,43,44] reported that, below pH value of 10.6, ettringite was no longer stable, and gypsum formed. Unlike the other samples, C-CON did not change the type of reaction product in the HCl solution, possibly due to the absence of ettringite and Ca(OH)_2_ before immersion. In addition, the weight loss occurred in the vicinity of 800 to 900 °C in CaO-activated concrete, which might be the recrystallization of C-S-H [45]. 

**Figure 10:**
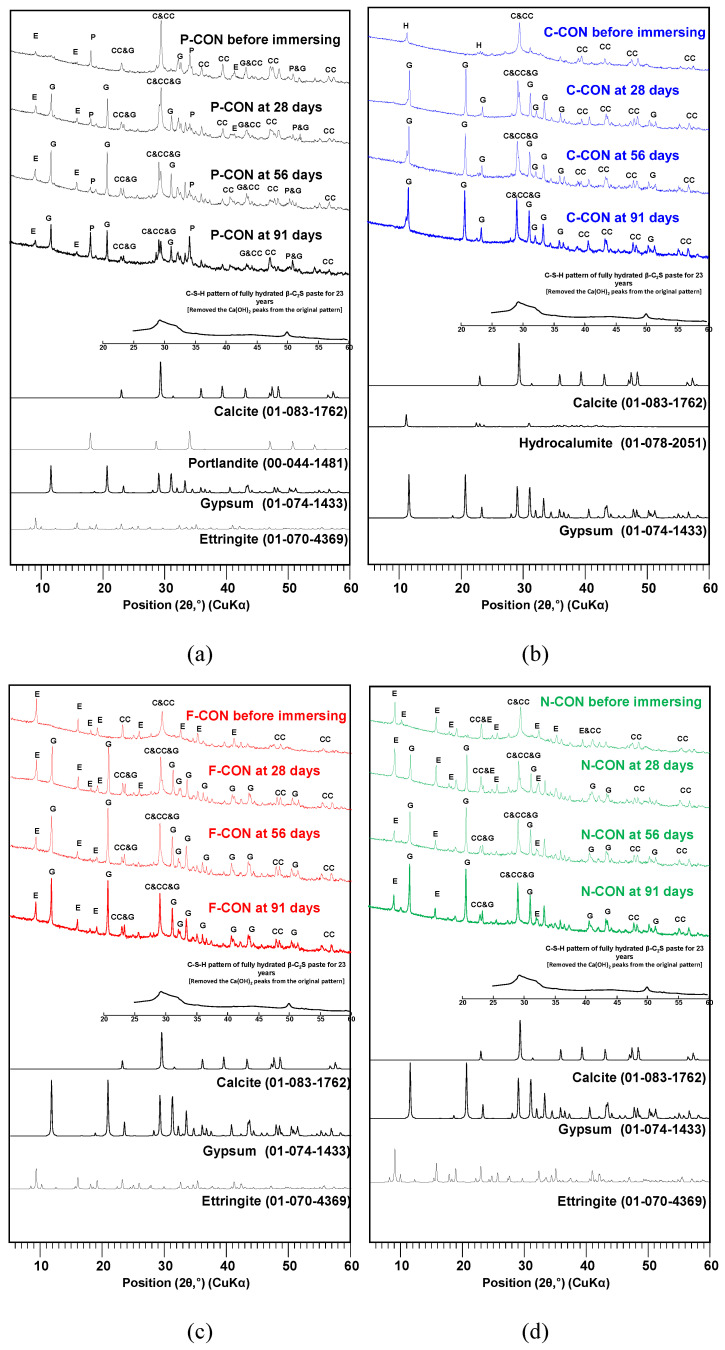
XRD patterns of hardened samples immersed in 5% H_2_SO_4_ solution: (**a**) P-CON, (**b**) C-CON, (**c**) F-CON, and (**d**) N-CON. (Note. E: ettringite, C: C-S-H, CC: calcite, G: gypsum, H: hydrocalumite, and P: portlandite, respectively).

**Figure 11:**
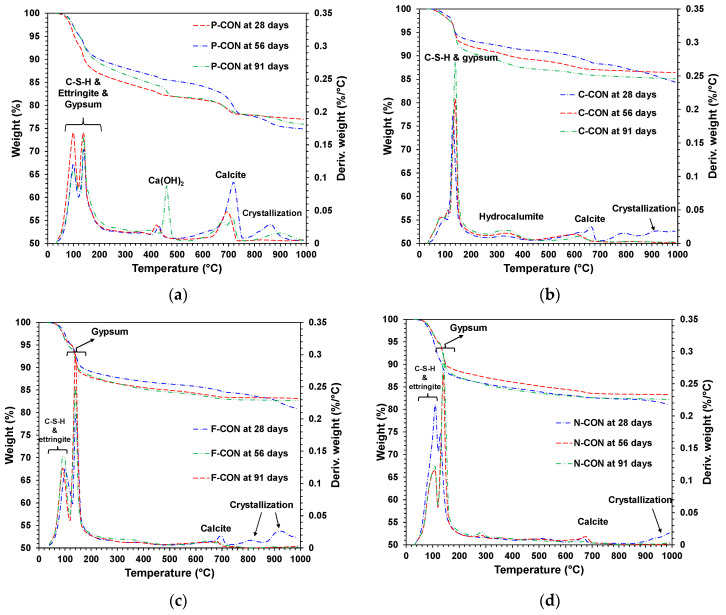
TG/DTG curves of hardened samples immersed in 5% H_2_SO_4_ solution: (**a**) P-CON, (**b**) C-CON, (**c**) F-CON, and (**d**) N-CON.

Figure 10 and Figure 11 show the results of XRD and TG/DTG tests, respectively, for the samples in the H_2_SO_4_ solution. In addition, similar to the HCl results, regeneration of Ca(OH)_2_ at the P-CON sample and crystallization were observed. It is worth noting that ettringite is relatively more susceptible to the drying process of sample preparation than other cementitious reaction products and, thus, ettringite can often be underestimated in quantity in XRD and TG/DTG [46,47]. Unfortunately, in this study, the result for ettringite in the H_2_SO_4_ solution in TG/DTG was not precisely consistent with that of XRD; however, careful interpretation enabled us to recognize the approximate tendency of the product change.

In the samples that had ettringite before immersion (i.e., P-, F-, and N-CON in Figure 10), as the DTG peaks of ettringite decreased, the gypsum DTG peaks tended to increase, as shown in Figure 11. In particular, the F-CON and N-CON samples showed relatively strong gypsum XRD and DTG peaks, and the peaks were even stronger than those of samples in the HCl solution. The cause of gypsum formation was similar to the case of HCl immersion; as the pH of the concrete was likely lowered in the H_2_SO_4_ solution, ettringite became unstable and decomposed, producing gypsum. 

However, note that gypsum can also form from the reaction between Ca(OH)_2_ and H_2_SO_4_ without ettringite decomposition [27,41]. For instance, in P-CON, because Ca(OH)_2_ was present before immersion, this reaction surely accounted for a portion of gypsum formation. However, this reaction was also likely responsible for the gypsum formation in C-CON despite no Ca(OH)_2_ before immersion (Figure 10b and Figure 11b). Previous studies [27,41,48,49,50,51] reported that C-S-H and calcite can dissolve, producing Ca^2+^ ions (or Ca(OH)_2_) when the pH is lowered. In the TG/DTG results, the decrease in calcite peak was—relatively—clearly seen, but the decrease in C-S-H peak was difficult to observe because the C-S-H peak below 200 °C overlapped with the peaks of ettringite and gypsum, making it challenging to analyze. However, when referring to the other study results [49,50,51], C-S-H likely dissolved to some extent similar to calcite. Thus, the dissolved Ca^2+^ ions can produce gypsum from the reaction with H_2_SO_4_ in C-CON. On the other hand, in the case of P-CON, the coexistence of Ca(OH)_2_ generally prevents chemical deterioration of C-S-H [49,52]; thus, a relatively smaller amount of C-S-H likely dissolved than the other samples. 

## 4. Conclusions

This study examined the mechanical and durability properties of three types of CaO-activated GGBFS concretes made with three different additives (CaCl_2_, Ca(HCCO)_2_, and Ca(NO_3_)_2_), and compared their properties with those of Portland cement concrete. Detailed conclusions are summarized as follows:

1. All CaO-activated GGBFS concretes satisfied the targeted air content and slump ranges. However, they exhibited lower mechanical properties (compressive strength, split strength, and elastic modulus) than OPC concrete.
2. In the CaO-activated GGBFS system, the additive CaCl_2_ was the most effective at increasing strength and elastic modulus, while the additive Ca(NO_3_)_2_ was the least effective.
3. In the freezing and thawing cycle test, the CaO-activated GGBFS concretes with added Ca(HCOO)_2_ and Ca(NO_3_)_2_ were destroyed before 300 cycles; however, the one made with CaCl_2_ had a relatively comparable resistance in the freezing and thawing cycles test to OPC concrete.
4. The strength was a less accurate indicator than the dynamic modulus for estimating the ability to resist freezing and thawing cycles in this study.
5. The chemical resistance tests using 5% HCl and 5% H_2_SO_4_ solutions showed significantly different results depending on the type of acid solutions. In a 5% HCl solution, although weight loss was observed in all immersed samples, the OPC concrete had the most excellent resistance, while the CaO-activated GGBFS concrete with Ca(NO_3_)_2_ was the most vulnerable. However, in 5% H_2_SO_4_ aqueous, the most considerable weight loss occurred in the OPC concrete.
6. In XRD, all concrete with added sulfate ion (SO_4_^2−^) (i.e., Portland cement concrete and CaO-activated GGBFS concretes with Ca(HCOO)_2_ and Ca(NO_3_)_2_) had ettringite before immersion. When they were immersed in HCl solution, their DTG results showed that ettringite tended to decrease, and gypsum was generated; however, the CaO-activated GGBFS concrete with CaCl_2_ did not change the type of reaction product in the HCl solution, possibly due to the absence of ettringite and Ca(OH)_2_ before immersion.
7. When immersed in H_2_SO_4_ solution, similar to the case of HCl, the DTG peaks of ettringite decreased, and the gypsum DTG peaks tended to increase. However, in the H_2_SO_4_ solution, gypsum was synthesized from the reaction between Ca(OH)_2_ and H_2_SO_4_ in all concrete samples. In particular, the concrete without ettringite (i.e., CaO-activated GGBFS concrete with CaCl_2_) had a considerable amount of gypsum formation; in this concrete, dissolved C-S-H and calcite, due to the low pH, likely produced Ca^2+^ ions, and gypsum formed from the reaction between Ca^2+^ and H_2_SO_4_.

## Figures and Tables

**Table 1:** Oxide compositions: OPC and GGBFS (wt%).

Oxide	OPC	GGBFS
CaO	64.05	59.48
SiO_2_	18.59	23.05
Al_2_O_3_	4.45	9.72
MgO	3.14	2.54
SO_3_	3.55	1.37
TiO_2_	0.29	0.98
MnO	0.19	0.83
Fe_x_O_y_	3.61	0.68
K_2_O	1.23	0.68
Na_2_O	0.29	0.28
Others	0.61	0.4

**Table 2:** Material proportions of binders used in concrete mixture samples (wt%).

Binder Type	OPC	GGBFS	CaO	CaCl_2_	Ca(HCOO)_2_	Ca(NO_3_)_2_	CaSO_4_	Sum
P-binder	100	0	0	0	0	0	0	100
C-binder	0	94	4	2	0	0	0	100
F-binder	0	88	4	0	3	0	5	100
N-binder	0	86	4	0	0	5	5	100

**Table 3:** Mixture proportions of concrete samples.

Sample	Binder Type	G_max_ (mm)	w/b (wt%)	s/a (vol%)	Unit Weight (kg/m^3^)				
					Water	Binder	FA	CA	Plasticizer
P-CON	P-binder	25	36.73	42.31	176.63	480.82	687.81	955.86	0.14
C-CON	C-binder						676.34	939.93	0.14
F-CON	F-binder						673.87	936.48	0.14
N-CON	N-binder						673.87	936.48	0.14

Note: FA denotes fine aggregate; CA denotes coarse aggregate; G_max_ denotes the ratios of the maximum size of coarse aggregate; w/b denotes water to binder weight ratio, and s/a denotes sand to aggregate volume.

**Table 4:** The results of elastic modulus using experiments and model codes.

Sample Label	Compressive Strength at 28 Days (MPa)	Density (kg/m^3^)	Elastic Modulus (GPa)		
			Exp.	ACI 318	CEB-FIP
P-CON	44.8	2415.5	28.8	34.1	41.5
C-CON	30.5	2372.6	20.9	27.4	35.7
F-CON	29.5	2349.7	19.1	26.6	34.7
N-CON	20.7	2360.2	17.2	19.7	29.1

## Data Availability

Not applicable.
